# Concordant and Heterogeneity of Single-Cell Transcriptome in Cardiac Development of Human and Mouse

**DOI:** 10.3389/fgene.2022.892766

**Published:** 2022-06-27

**Authors:** Mengyue Shang, Yi Hu, Huaming Cao, Qin Lin, Na Yi, Junfang Zhang, Yanqiong Gu, Yujie Yang, Siyu He, Min Lu, Luying Peng, Li Li

**Affiliations:** ^1^ Key Laboratory of Arrhythmias, Ministry of Education of China, Shanghai East Hospital, Tongji University School of Medicine, Shanghai, China; ^2^ Heart Health Center, Shanghai East Hospital, Tongji University School of Medicine, Shanghai, China; ^3^ Institute of Medical Genetics, Tongji University, Shanghai, China; ^4^ Department of Cardiology, Shanghai Shibei Hospital, Shanghai, China; ^5^ Department of Medical Genetics, Tongji University School of Medicine, Shanghai, China; ^6^ Research Units of Origin and Regulation of Heart Rhythm, Chinese Academy of Medical Sciences, Beijing, China

**Keywords:** single-cell RNA sequencing, cross-species comparison, embryonic heart development, gene transcription, cardiomyocytes

## Abstract

Normal heart development is vital for maintaining its function, and the development process is involved in complex interactions between different cell lineages. How mammalian hearts develop differently is still not fully understood. In this study, we identified several major types of cardiac cells, including cardiomyocytes (CMs), fibroblasts (FBs), endothelial cells (ECs), ECs/FBs, epicardial cells (EPs), and immune cells (macrophage/monocyte cluster, MACs/MONOs), based on single-cell transcriptome data from embryonic hearts of both human and mouse. Then, species-shared and species-specific marker genes were determined in the same cell type between the two species, and the genes with consistent and different expression patterns were also selected by constructing the developmental trajectories. Through a comparison of the development stage similarity of CMs, FBs, and ECs/FBs between humans and mice, it is revealed that CMs at e9.5 and e10.5 of mice are most similar to those of humans at 7 W and 9 W, respectively. Mouse FBs at e10.5, e13.5, and e14.5 are correspondingly more like the same human cells at 6, 7, and 9 W. Moreover, the e9.5-ECs/FBs of mice are most similar to that of humans at 10W. These results provide a resource for understudying cardiac cell types and the crucial markers able to trace developmental trajectories among the species, which is beneficial for finding suitable mouse models to detect human cardiac physiology and related diseases.

## 1 Introduction

The heart is the first-formed organ in embryonic development (Sylva et al., 2014; Asp et al., 2019). The development of the embryonic heart involves a complex process in which the organ is continually remodeled as chambers are formed, valves are sculpted, and connections are established to the developing vascular system (Barnett et al., 2012). The normal function of the mammalian heart is inseparable from the interaction of different cells in the organ, such as cardiomyocytes (CMs), fibroblasts (FBs), endothelial cells (ECs), and epicardial cells (EPs). The differentiation of these cells with precise spatiotemporal regulation is also necessary during the development to establish the heart structure (Meilhac and Buckingham, 2018). Dissecting the molecular details of human heart development is an important prerequisite for developing cardiac regenerative medicine, generating disease models and exploring potential therapeutic targets. However, due to some limitations, such as rarity and ethics, it is quite difficult to use human specimens for analyzing early cardiac development. In recent years, mice have been widely used as an alternative model to explore molecular regulation in development.

ScRNA-seq analysis using heart tissue has allowed us to explore various cell types for the cardiovascular system, significantly contributing to our understanding of the spatial and temporal expression patterns of these cell types and the heterogeneity within their subpopulations (Dalerba et al., 2011; Gawad et al., 2014; Giustacchini et al., 2017; Li et al., 2017; Briggs et al., 2018; Ledergor et al., 2018; Plass et al., 2018). The single-cell transcriptome data of heart cells from embryonic day 8.5 to postnatal day 21 in mice have revealed the transcriptional characteristic for each cell type during the process, in which the deletion of Nkx-2.5 leads to severe defects in cardiomyocyte maturation, accompanied by significant heterogeneity in known cardiomyocyte cell types (DeLaughter et al., 2016; Li et al., 2016). Moreover, the cellular maps with organ development at days 9.5 and 13.5 of mouse gestation have been established (Cao et al., 2019). Single-cell landscape maps of 20 adult mouse organs or tissues, including the heart, have also been generated (The Tabula Muris Consortium, 2018). Analysis of cardiac single-cell data from four critical periods of mouse embryonic heart development revealed that congenital heart malformations are mainly due to defects of cell sources from the second heart field (Xiong et al., 2019). Single-cell sequencing data have also been used to provide insight into the key regulatory mechanisms involved in the specialization of progenitor cells to multiple lineages during early cardiac development, for example, how Hand2 plays the role as a key specialization factor of the outflow tract (de Soysa et al., 2019). Recently, a human cardiac cell atlas has been constructed to understand the development of the organ (Litvinukova et al., 2020). More progress includes generating the first human developing heart model with a single-cell spatial resolution (Asp et al., 2019), systematically resolving the unique biological features of the major cell types with single-cell resolution, revealing the spatiotemporal-specific activation of key signaling pathways and the complex signaling interactions between cardiomyocytes and non-cardiomyocytes (Cui et al., 2019). However, the differences in cellular characteristics of embryonic development of mouse and human hearts remain incompletely understood. Further comparative analysis of the patterns of different lineages’ fate in mouse and human heart development will be beneficial for fully understanding the details of heart development.

In the present work, we integrated datasets from different data sources to explore lineage-specific changes in expression profiles, subpopulation composition, and developmental trajectories in cardiac tissues of both species by comparing human and mouse developing hearts data. We identified marker genes that are shared and specific in different cell types between human and mouse hearts and also analyzed the most similar time points between the different species in the process, which provides a reliable framework for comprehending human heart development using a mouse model.

## 2 Materials and Methods

### 2.1 Data Access

The GSE106118 dataset in the GEO database (https://www.ncbi.nlm.nih.gov/geo/) contains human embryonic heart development data using STRT-seq. A total of 4,948 cells from 20 human fetal hearts were collected from 5 W to 25 W of gestation (Cui et al., 2019). Mouse dataset1 (GSE76118) was obtained using Smart-seq2 and contained the data of three time points: e8.5, e9.5, and e10.5, with a total of 2,967 cells (Li et al., 2016); two time points of mouse embryonic heart development: e13.5 and e14.5, a total of 11,532 cell data were obtained, which was named mouse dataset2 (GSE100861) (Xiao et al., 2018). The above data were imported into the Seurat v4.0.6 R package (https://satijalab.org/seurat/) for stringent quality control.

### 2.2 Data Quality Control and Integration

To avoid the impact of low-quality data on subsequent analyses and to maximize the retention of important and rare cell subpopulations or transcripts, we observed the distribution of samples, eliminated outliers, and retained as many transcripts or cells as possible. For cell filtering, the number of captured transcripts per gene of human embryonic hearts was inferred based on UMIs. Only cells with a minimally detected gene number of 1,000 were kept for downstream analysis. The number of transcripts detected less than 5,000 were also filtered. For mouse dataset1, only cells with the number of genes detected greater than 1,800 and the number of transcripts detected at least 1E + 06 were kept. Mouse dataset2 was processed with the same strategy. In addition, the proportion of mitochondrial genes to all genetic material may indicate whether a cell is in homeostasis or not. The cells with high proportions of mitochondrially-derived genes might indicate damaged or dead since the cell could lead to leakage of cytoplasmic RNA molecules, while mRNA in the mitochondria might be retained. Therefore, cells with high mitochondrial gene expression need to be filtered (Luecken and Theis, 2019; Andrews et al., 2021). In the heart, about 30% of the total mRNA comes from mitochondria due to the high energy demand of cardiomyocytes (Mercer et al., 2011; Galow et al., 2021). Thus, the cells with a mitochondrial gene percentage of >30% were moved out in both species. DoubletFinder was used to identify and remove doublets (McGinnis et al., 2019). Since the two mouse datasets were generated using different sequencing technologies (Drop-seq and Smart-seq2), the different sequencing depths and normalization means prevented the two datasets from being directly combined for analysis. A geometry-based sampling of mouse dataset2 from the Drop-seq platform was performed using Python’s geosketch to ensure equilibrium between the two mouse datasets. This geometry-based sampling approach was able to retain rare cell types and accelerated the integration of large scRNA-seq datasets, thereby improving and accelerating downstream analysis. 2,402 cells were finally sampled from mouse dataset2. For each dataset, the counts of each cell were normalized using the “NormalizeData” function in Seurat with a scale factor of 10,000. ScaleData function was used then for standardization. The top 2000 highly variable genes were selected using the FindVariableFeatures function. To correct potential batch effects in mouse datasets from different platform sources, we integrated them using the R package Harmony (https://github.com/immunogenomics/harmony). In the analysis, following the examples provided in the Harmony package in the R language environment, we performed Harmony within the Seurat 2 workflow with the maximal number of clusters (50) and the maximal number of iterations (100). The top 20 normalized Harmony vectors in PCA space were used as input to the assessment methods.

### 2.3 Identification of Cell Types

The pre-processed data were unsupervised and clustered using the FindClusters function in Seurat to identify different cell types based on the abundance of cell subpopulations and the representation of cell subpopulations in cardiac function based on known marker genes. Cells were classified into 20 clusters for humans and 17 clusters for mice. Next, we used the FindAllMarkers function to find differentially expressed genes (DEGs) of each type of cell.

### 2.4 Cross-Species Comparison

The cross-species comparison was based on one-to-one orthologues annotated by the Ensembl genome annotation system (http://www.ensembl.org/index.html). The average expression levels in major cell types were calculated. Pearson correlations of major cell types in humans and mice were used to determine cross-species similarities.

### 2.5 Trajectory Analysis

Reconstructing pseudotime trajectories of cells were constructed using the Monocle2 R package (version 2.18.0, http://cole-trapnell-lab.github.io/monocle-release/) (Trapnell et al., 2014). First, the UMI of CMs, FBs, and ECs/FBs were respectively imported into Monocle2. Genes that were expressed in at least 10 cells were used, and only genes expressed in 5% of cells were kept. We used the differentially expressed genes between time points as the set of ordering genes and performed the dimension reduction and the trajectory analysis. Once we established a trajectory, we used the differentialGeneTest function to find genes that had an expression pattern that varied according to pseudotime.

### 2.6 Gene Ontology (GO) Enrichment Analysis

GO characteristics of gene clusters were determined using the clusterProfiler package (v3.10.0.94) (Yu et al., 2012) for all DEGs with an average logFC value above zero and an adjusted *p-*value below 0.01.

### 2.7 Identification for Human–Mouse Time Point Similarity

According to a previous work (DeLaughter et al., 2016), PCA profiles for each time point of the cell types in human hearts were produced first and a density map based on the principal component PC1 that could better reflect the developmental stage was generated. Then, the loading value of PC1 was calculated. After that, the loading value and the expression of homologous genes in mouse corresponding cell types were used to calculate the PC1 value of the mouse. Finally, the PC1 values of the mouse were mapped to the human density plot.

## 3 Results

### 3.1 Pre-Processing of Data and Integration of Mouse Datasets From Different Platforms

Stringent quality control was performed using Seurat on the abovementioned three datasets (Supplementary Figures S1A–F). 3,757, 2,402, and 9,757 high-quality cells were further analyzed (Satija et al., 2015). To ensure the integrity of the mouse dataset at time points and comparability with the human dataset, the two mouse datasets were then sampled and integrated. We first used a geometry-based sampling approach for mouse dataset2, from which we took a comparable number of cells to dataset1 for subsequent integration (Hie et al., 2019) (Supplementary Figure S1G). After using Harmony integration (Korsunsky et al., 2019), cells from both datasets were overlapped well together (Supplementary Figure S1H).

### 3.2 Identification of Cell Types and Cell-Type–Specific Genes in Human and Mouse

**FIGURE 1:**
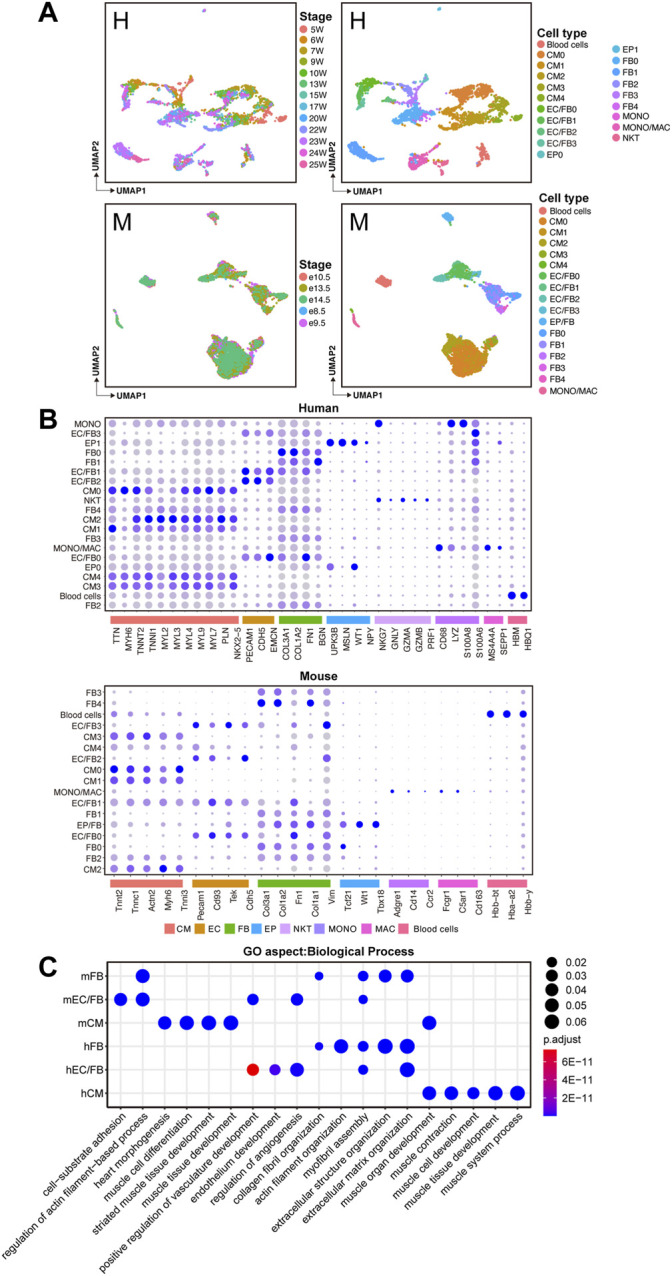
Identification of major cell types in human and mouse fetal hearts. **(A)**UMAP plots showing the developmental stages (left) and cell clusters (right) of human (H, upper) and mouse (M, lower) fetal heart development. **(B)** Bubble plots showing the expression levels of known marker genes for each cell population. **(C)** GO enrichment analysis of differentially expressed genes between human and mouse cell types.

Here, we used the same software and strategies to analyze the human and mouse datasets. After dimensional reduction, doublets removal, and unbiased clustering, 20 cell clusters were identified in human hearts. According to the expression status of marker genes, we further classified cells into major cell types, including 5 cardiomyocyte clusters (CMs) with high expression of cardiomyocyte-specific marker genes, such as *TTN*, *MYH6*, and *TNNT2*; 5 fibroblast clusters (FBs) with high expression of fibroblast-specific marker genes, such as *COL3A1*, *COL1A2,* and *FN1*; 4 endothelial cell/fibroblast clusters (ECs/FBs) with co-expression of endothelial cell-specific marker genes (NKX2-5, *CDH5*, and *EMCN*) and fibroblast-specific marker genes; 2 epicardial cell clusters (EPs) with high expression of epicardial cell-specific marker genes (*UPK3B, MSLN, and WT1*); macrophage cluster (MACs) with high expression of *MS4A4A* and *SEPP1*; macrophage/monocyte cluster (MACs/MONOs) with high expression of both macrophage marker genes and monocyte marker genes (*CD68, LYZ, S100A8, and S100A*6); natural killer cells cluster (NKTs) with high expression of *NKG7, GNLY,* and a cluster of blood cells (Figures 1A and B). A total of 17 clusters were identified in mouse hearts, including 5 CM clusters with high expression of *Tnnt2, Tnnc1, and Actn2*; 5 FB clusters with high expression of *Col3a1, Col1a2, and Fn1*; 4 EC/FB clusters co-expressing endothelial cell maker and fibroblast marker, such as *Pecam1, Cd93, Tek, and Cdh5*; 1 epicardial/fibroblast cluster (EPs/Fibroblasts) and a cluster of MACs/MONOs and a blood cells cluster (Figures 1A and B). Major cell types, including CMs, FBs, ECs, and immune cells (MONOs/MACs), were identified in both human and mouse hearts, indicating overall relative conservation of cell types between the two species.

Based on the defined cell types, we further identified cell-type–specific genes by performing differential expression analysis between each cell type and the others. Then GO enrichment analysis (biological process, BP) was performed on the DEGs (Supplementary Table S1). As shown in Figure 1C, in the two species, the cell-type-specific genes of CMs were mainly enriched with muscle tissue development and cardiac morphogenesis. Cell-type-specific genes of FBs were mainly associated with the organization of extracellular matrix, actin filament, and collagen fibril. In the EC/FB populations, in addition to functions such as extracellular matrix organization and cell-substrate adhesion, some functions such as angiogenesis regulation and positive regulation for vasculature development were also enriched. Since FBs exert an important role in cardiac development through the deposition of collagen and other extracellular matrix (ECM) components, and ECs involve maintaining vascular homeostasis and cardiac function, the functional enrichment analysis shows a well-characterized biological profile for each cell type in this work.

### 3.3 Concordance and Inconsistency in Cell Type in Humans and Mice

**FIGURE 2:**
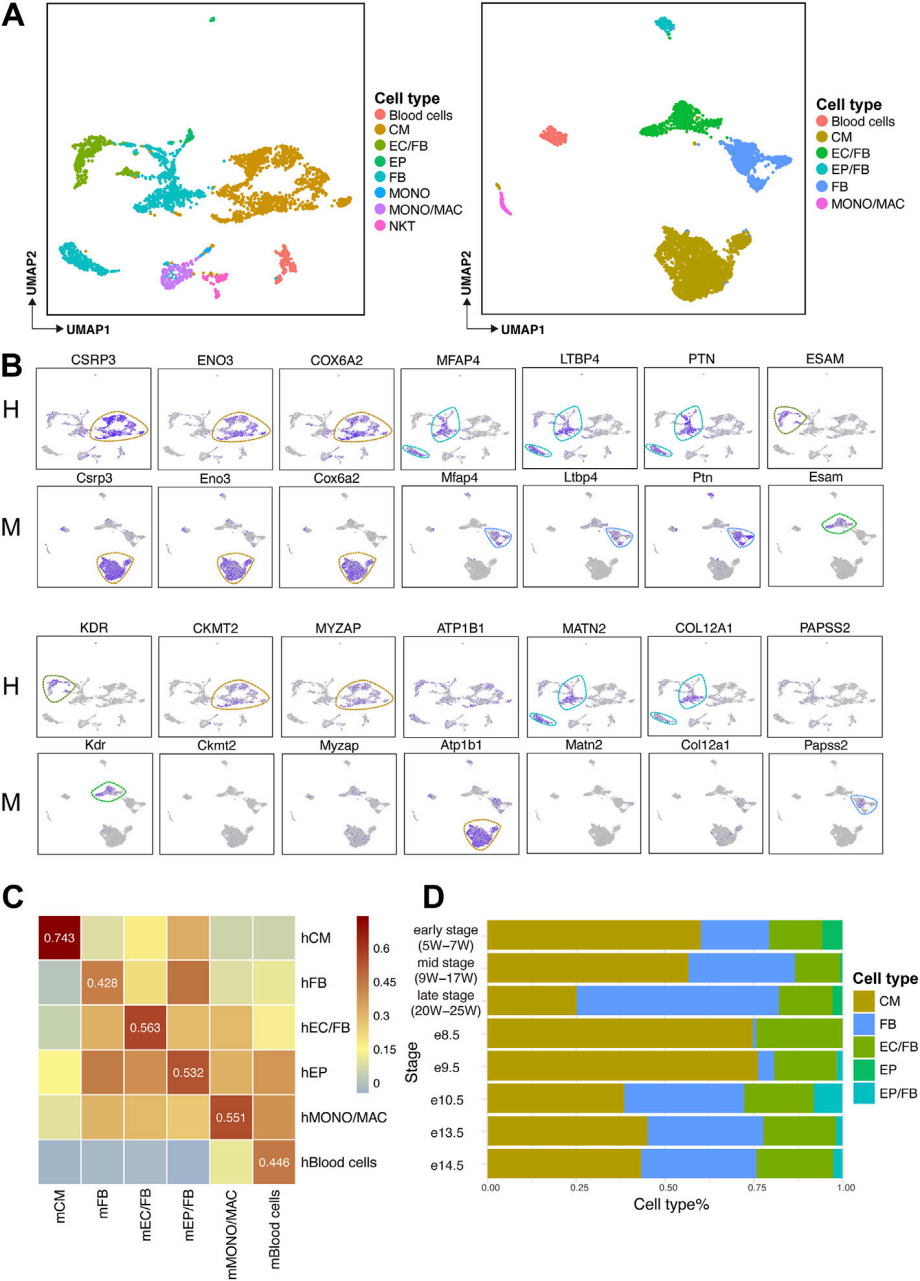
Expression levels of marker genes in human and mouse heart and comparison of cell type correlation and cell type composition ratio across species. **(A)** UMAP plots showing the cell types of human (H, left) and mouse (M, right) fetal heart development. **(B)** UMAP plots showing the expression levels of each marker gene in human (H) and mouse (M). **(C)** Heatmaps showing the Pearson correlations of major cell types between human (H) and mouse (M) fetal heart. **(D)** Bar plot showing the cell composition at each stage of human and mouse heart development.

Based on the defined cell types in human and mouse (Figure 2A), we further explored the expression of marker genes in each cell type in both species. We found that some previously-unreported genes conserved expression in both human and mouse, such as *CSRP3*, *ENO3,* and *COX6A2* in CMs; *PTN*, *LTBP4,* and *MFAP4* in FBs; *KDR* and *ESAM* in ECs/FBs. Meanwhile, some species-specific marker genes, such as *CKMT2* and *MYZAP*, which were only expressed in human CMs, and *ATP1B1* was only expressed in mouse CMs. As for FBs, *MATN2,* and *COL12A1* genes were highly expressed only in humans, while *PAPSS2* expression showed a specificity with mouse (Figure 2B).

To understand whether the human concordant cell types are similar to mouse ones, an analysis based on homologous genes was performed. We found a high correlation between cardiomyocytes population, indicating that identical cell types in two species are more relevant than non-identical ones (Figure 2C).

Next, we observed the composition of the major cell types at different developmental stages in human and mouse hearts. The result showed that the proportion of CMs was greatly reduced, but the proportion of non-cardiomyocytes was increased in late developmental stages compared to early developmental stages (Figure 2D).

### 3.4 Developmental Patterns and Corresponding Similar Developmental Stages in Human and Mouse Cardiomyocytes

**FIGURE 3:**
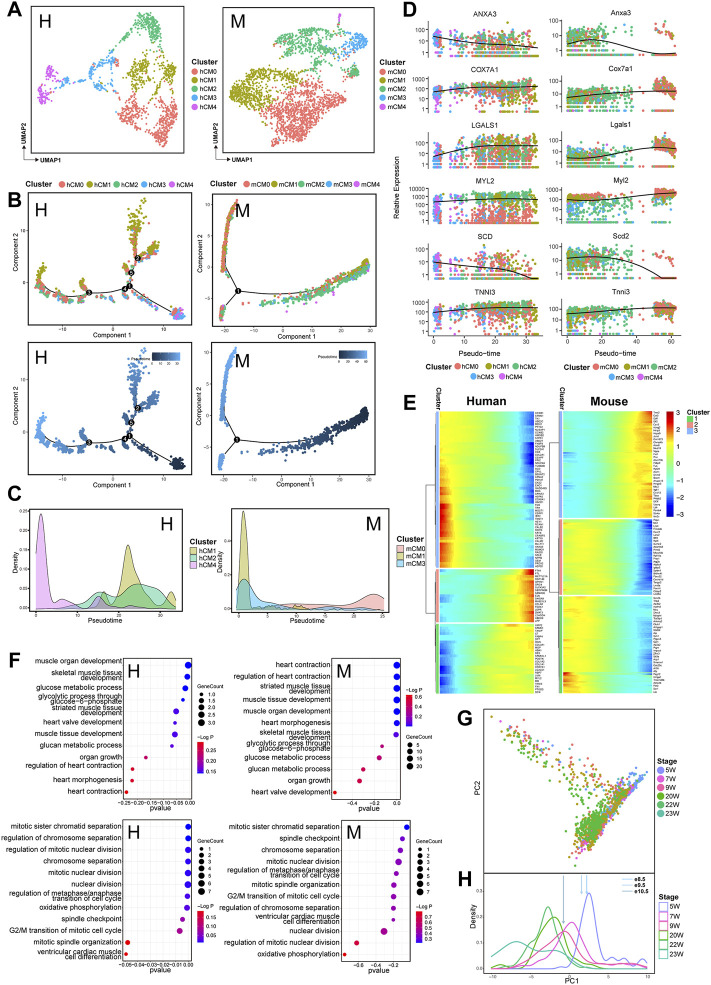
Transcriptomic dynamics of cardiomyocytes during human and mouse heart development (human: H, left; mouse: M, right). **(A)** UMAP plots showing the subpopulations of human (H) and mouse (M) cardiomyocytes. **(B)** Pseudotemporal trajectory of CM subpopulations constructed by Monocle2. Upper: colored by CM subpopulations; Lower: colored by pseudotime values, with the value ranging from small to large representing the start to the end of the developmental process. **(C)** Density plot of the distribution of CM subpopulations along the trajectory. **(D)** Dot plots showing the expression of genes conserved along the trajectory in two species. **(E)** Heatmap showing the expression pattern of cardiomyocytes along the developmental trajectory. **(F)** GO enrichment analysis (BP) of the genes in cardiomyocytes with different expression patterns along the trajectory between two species. **(G)** PCA plot of cardiomyocyte expression at various time points in human hearts. **(H)** PCA-based density plot depicting the proportion of hCMs at various time points, showing a considerable overlap in temporal development from 5 W to 24 W. The arrows represent the mean expression of different developmental stages of mouse CMs.

In order to further understand the conservation and differences of cellular composition and gene expression pattern at various developmental stages of human and mouse hearts, a comparative analysis of gene expression was applied at different time points for each cell type in both species. Although the process of heart development is synergized by multiple cell lineages, the function of cardiomyocytes is more crucial to maintain heart development (Kelly et al., 2014). Therefore, we first further subpopulated the cardiomyocyte population. An unbiased clustering was used to classify cardiomyocytes into five subpopulations: CM0, CM1, CM2, CM3, and CM4 (Figure 3A, Supplementary Figure S2A). To figure out the progressive molecular changes of CMs during the maturation process, we performed a pseudotime analysis on these cardiomyocyte subpopulations to reconstruct the trajectory of cardiomyocyte development (Figure 3B, Supplementary Figure S2B). In this process, only the genes expressed by at least 5% of the CMs were retained. We used the DEGs between time points as sorted gene sets for dimension reduction and trajectory analysis. For both human and mouse CMs, a pseudotime trajectory was constructed starting from 5 to 9 W (human) and e8.5-e9.5 (mouse) cells and gradually progressing to 23–25 W (human) and e13.5-e14.5 (mouse) cells, respectively. Since selected the dataset with developmental stage information for cell labeling, we found that the pseudotime trajectories colored by developmental time points could reasonably reflect the process of CMs development (Figure 3B, Supplementary Figure S2B). In order to investigate the potential role of CMs subpopulations in their development and in their functional stage, we next observed the expression profile of different CMs subpopulations in the pseudotime trajectory. The density curves of cell subpopulation expression generated from the trajectory showed that in human CMs, hCM4 subpopulation was aligned at the beginning of the trajectory, whereas hCM1 and hCM2 ones mainly appeared at the end of the trajectory. While both mCM1 and mCM3 subpopulations were aligned at the start point of the trajectory and mCM0 one was presented around the trajectory end (Figure 3C), suggesting that hCM4, mCM1, and mCM3 may exert their function in early cardiac development, while hCM1, hCM2, and mCM0 may play a role in later cardiac development. Along with heart development, a series of genes with conserved expression patterns were detected in cardiomyocyte subpopulations (Figure 3D), for example, *TNNI3* and *MYL2* remain highly expressed, *LGALS1* and *COX7A1* were significantly and progressively upregulated, and both *ANXA3* and *SCD* were found to be downregulated along the trajectory. Consistent with this, the subpopulations of hCM4, mCM1, and mCM3 that were arranged at the beginning of the developmental trajectory also show relatively high expression of *ANXA3* and *SCD*; the subpopulations of hCM1, hCM2, and mCM0 that were arranged at the end of the developmental trajectory also had relatively high expression of *LGALS1* (Supplementary Figure S2C). These significantly expressed genes are really crucial in maintaining heart development. For example, *LGALS1*, as a member of the highly conserved β-galactoside-binding lectin family, has been identified to play an essential role in the control of inflammation and neovascularization (Camby et al., 2006; Liu and Rabinovich, 2010). *Gal-1* encoded a major component of the contractile apparatus of cardiomyocytes involved in the regulation of cardiac function (Dias-Baruffi et al., 2010). As a mitochondrial function-related gene, *COX7A1* is necessary for both systolic and diastolic functions (Huttemann et al., 2012). In addition, *ANXA3* can protect the myocardium from ischemic injury and reduce infarct size (Meng et al., 2019). *SCD* encodes a central enzyme that synthesizes monounsaturated fatty acids in lipid metabolism. Recent evidence shows that *SCD* can reprogram cardiac metabolism, thereby regulating cardiac function (Dobrzyn et al., 2015). In the heart, *SCD1* deficiency enhances glucose transport and metabolism at the expense of fatty acid uptake and oxidation (Izquierdo-Garcia et al., 2018). The metabolic changes associated with *SCD1* deficiency really protect cardiac myocytes against both necrotic and apoptotic cell death and improve heart function.

Based on the developmental trajectory of CMs, we divided the affected-trajectory genes into three gene clusters that are highly expressed at the initial, transitional, and terminal stages of the trajectory. The functional enrichment of different gene clusters closely reflects the molecular signature of CMs from the immature to the mature state (Figure 3E, Supplementary Table S2). GO analysis revealed that in both human and mouse hearts, high expression at the beginning of the trajectory gene (HEBTG) was enriched in functions such as regulation of mitotic nuclear division and cell cycle (Figure 3F, Supplementary Table S2), while high expression at the end of the trajectory gene (HEETG) was enriched in the functions, including muscle system development and muscle contraction (Figure 3F) (Jouandin et al., 2014).

A temporal evolution of CM gene expression has been analyzed in both ventricular and atrial cardiomyocytes in mouse from e9.5 to P21 (DeLaughter et al., 2016). In a similar way, we also observed a temporal evolution of gene expression in hCMs through PCA analysis at all time points based on the genes expressed in at least 75% of hCMs (Figure 3G). PCA component PC1 separated cells in overlapping, stepwise clusters ordered by age. To identify the most similar time points in human and mouse CMs, a density map was generated according to a reported method (DeLaughter et al., 2016) from the most informative components based on PCA (Figure 3H). MCMs gene expression in different developmental stages was then mapped to the human density plot using genes expressed in at least 75% of mCMs. We found that the expression data from e8.5 mCMs corresponded to 5 W hCMs, while data from e10.5 mCMs corresponded to 9 W hCMs (Figure 3H).

### 3.5 Developmental Patterns and Corresponding Similar Developmental Stages in Both Human and Mouse Fibroblasts Cells

**FIGURE 4:**
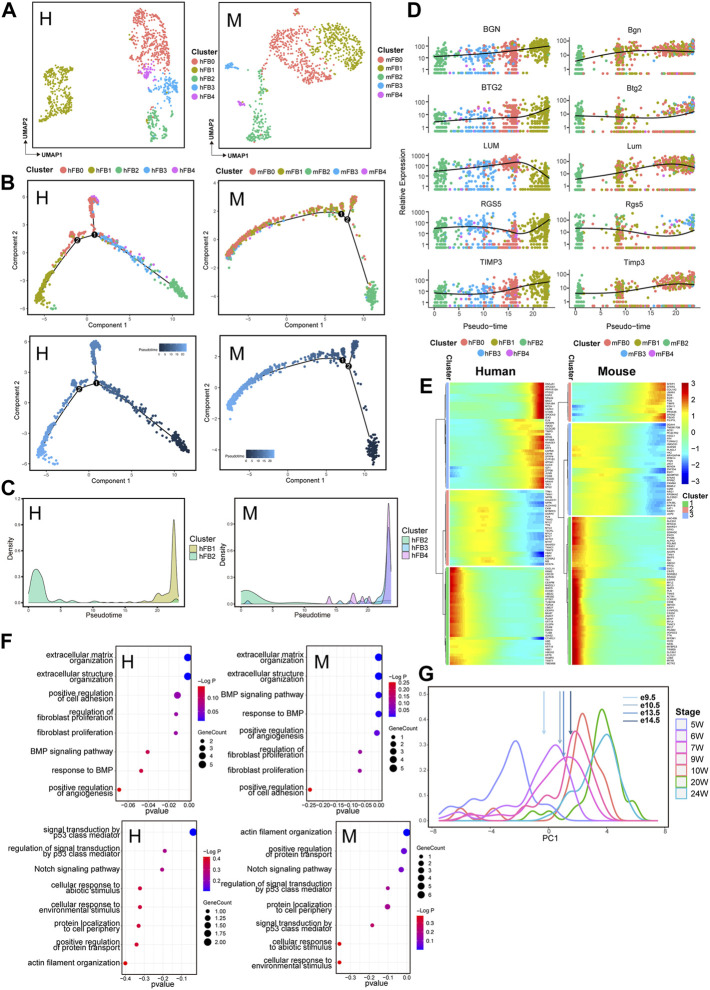
Transcriptomic dynamics of fibroblasts during human and mouse heart development (human: H, left; mouse: M, right). **(A)** UMAP plots showing the subpopulations of human (H) and mouse (M) fibroblasts. **(B)** Pseudotemporal trajectory of FB subpopulations constructed by Monocle2. Upper: colored by FB subpopulations; Lower: colored by pseudotime values, with the value ranging from small to large representing the start to the end of the developmental process. **(C)** Density plot of the distribution of FB subpopulations along the trajectory. **(D)** Dot plots showing the expression of genes conserved along the trajectory in two species. **(E)** Heatmap showing the expression pattern of fibroblasts along the developmental trajectory. **(F)** GO enrichment analysis (BP) of the genes in fibroblasts with different expression patterns along the trajectory between two species. **(G)** PCA-based density plot depicting the proportion of hFBs at various time points, showing a considerable overlap in temporal development from 5 W to 24 W. The arrows represent the mean expression of different developmental stages of mouse FBs.

Cardiac FBs have central effects on normal cardiac physiology and cardiovascular disease. The cells also play an important role in development *via* depositing collagen and other extracellular matrix (ECM) components (Tallquist, 2020). Therefore, we further analyzed the dynamics of the cell transcriptome during cardiac development. Unbiased clustering was utilized to classify human and mouse fibroblasts into five subpopulations: FB0, FB1, FB2, FB3, and FB4 (Figure 4A, Supplementary Figure S3A). The trajectory of FBs development was reconstructed to characterize the progressive molecular changes in the cells with the process (Figure 4B, Supplementary Figure S3B). Similarly, for human and mouse FBs, a temporal trajectory was constructed for each cell from early developmental stages to the later stages. To analyze the potential effects of different FBs subpopulations at the stages of fibroblast development, we next observed the expression status of different FBs subpopulations in the pseudotime trajectory. In hFBs, subpopulation hFB2 was aligned at the beginning of the trajectory, while subpopulation hFB1 appeared mainly at the end of the trajectory. For mFBs, mFB2 subpopulation was aligned at the beginning of the trajectory, and subpopulations of mFB3 and mFB4 appeared mainly at the end of the trajectory (Figure 4C). We also found that some genes with conserved expression patterns in FB subpopulations, such as *BTG2*, *RGS5,* and *TIMP3*, were significantly upregulated along the developmental process (Figure 4D). Consistently, subpopulations hFB1, mFB3, and mFB4 arranged at the end of the developmental trajectory also showed relatively high expression of *RGS5* and *BTG2* (Supplementary Figure S3C). *BTG2* encode an anti-proliferative protein of the BTG/Tob family that has been proved to inhibit proliferation and promote terminal differentiation of neurons (Farioli-Vecchioli et al., 2009) and skeletal muscle progenitors (Feng et al., 2007). *RGS5* can promote arterial growth during arteriogenesis (Arnold et al., 2014), and *TIMP3* may stimulate fibroblast proliferation and phenotypic differentiation into myofibroblasts at cardiac tissue injury sites (Lovelock et al., 2005). Also, a series of genes, such as *LUM*, showed a trend of being first being upregulated and then downregulated during both human and mouse heart development. (Figure 4D). Lumican (*LUM*) localized to the ECM as a keratan sulfate small leucine-rich proteoglycan that directly binds to fibrillar collagen and regulates collagen fibrillogenesis production in some tissues such as cornea, tendon, and skin, also is a central pro-fibrotic molecule in the heart (Mohammadzadeh et al., 2020). Moreover, we next divided the genes affecting the FBs development into three gene clusters that are highly expressed at the initial, transitional, and terminal stages of the trajectory (Figure 4E, Supplementary Table S3). The GO analysis of DEGs of FBs along the trajectory showed that HEETG was enriched in the function of extracellular matrix organization in both human and mouse hearts (Figure 4F, Supplementary Table S3), while HEBTG was enriched with cell cycle function. It should be noted that, HEBTG was also enriched in Notch and p53 signaling pathways, while HEETG was enriched in the BMP signaling pathway. These results suggest that the functional enrichment of DEGs along with trajectories closely reflects the molecular features of progressive maturation for FBs.

We next extended our inspection to FBs to observe the corresponding developmental stages of human and mouse hearts using the same strategy as CMs. We found that the expression data from e10.5 mFBs corresponded to 6 W hFBs, the data of e13.5 mFBs corresponded to 7 W hFBs, and the e14.5 -mFBs data corresponded to 9W hFBs, respectively (Figure 4G).

### 3.6 Developmental Pattern of a Mixed Subpopulation of EC/FB in Human and Mouse

**FIGURE 5:**
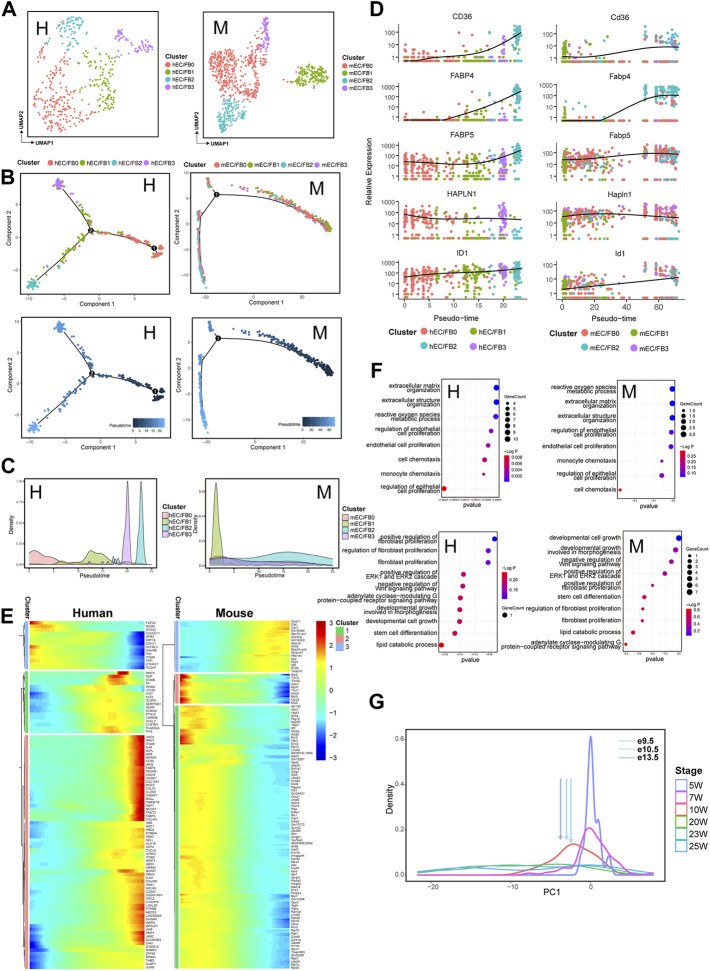
Transcriptomic dynamics of endothelial cells/fibroblasts during human and mouse heart development (human: H, left; mouse: M, right). **(A)** UMAP plots showing the subpopulations of human (H) and mouse (M) endothelial cells/fibroblasts. **(B)** Pseudotemporal trajectory of EC/FB subpopulations constructed by Monocle2. Upper: colored by EC/FB subpopulations; Lower: colored by pseudotime values, with the value ranging from small to large representing the start to the end of the developmental process. **(C)** Density plot of the distribution of EC/FB subpopulations along the trajectory. **(D)** Dot plots showing the expression of genes conserved along the trajectory in two species. **(E)** Heatmap showing the expression pattern of endothelial cells/fibroblasts along the developmental trajectory. **(F)** GO enrichment analysis (BP) of the genes in endothelial cells/fibroblasts with different expression patterns along the trajectory between two species. **(G)** PCA-based density plot depicting the proportion of hECs/FBs at various time points, showing a considerable overlap in temporal development from 5 W to 24 W. The arrows represent the mean expression of different developmental stages of mouse ECs/FBs.

ECs are crucial players in maintaining vascular homeostasis and cardiac function. We identified EC/FB clusters that express both endothelial and fibroblast markers in both human and mouse. Unbiased clustering was applied to divide the human and mouse EC/FB populations into four subpopulations: EC/FB0, EC/FB1, EC/FB2, and EC/FB3 (Figure 5A; Supplementary Figure S4A). Similarly, we also constructed a temporal trajectory for human and mouse ECs/FBs each with a progression from early to later developmental stages (Figure 5B; Supplementary Figure S4B). In hECs/FBs and mECs/FBs, subpopulations of hEC/FB0 and mEC/FB1 were alighted mainly at the beginning of the trajectory, while subpopulations of hEC/FB2, hEC/FB3, and mEC/FB2 were arranged at the end of the trajectory, suggesting that hEC/FB0 and mEC/FB1 may play a role at early stage of heart development; whereas hEC/FB2, hEC/FB3, and mEC/FB2 may have a function at a slightly later stage during the development (Figure 5C). A series of genes showed conserved expression patterns in the EC/FB subpopulation during the development (Figure 5D). For example, the expression of *CD36*, *FABP4*, and *ID1* was gradually upregulated during cardiac development, while genes such as *FABP5* and *HAPLN1* remained highly expressed throughout the development. Consistently, subpopulations hEC/FB2 and mEC/FB2 arranged at the end of the developmental trajectory also had relatively high expression of *CD36* and *FABP4* (Supplementary Figure S4C). These significant-expression genes are involved in many physiological functions, for example, *CD36* is closely related to fatty acid transport (Brinkmann et al., 2002), *FABP4* plays a key role in fatty acid uptake in the heart and skeletal muscle (Iso et al., 2013), and the ID family (including *ID1*) is essential for early heart formation. These results indicate that the representative subgroups hEC/FB2, hEC/FB3, and mEC/FB2 in both human and mouse really exert important effects in late cardiac development, especially in energy metabolism. Moreover, *HAPLN1* is an important regulator of ECM interactions during development (DeLaughter et al., 2013), and its expression pattern during cardiac development is consistent with extracellular matrix remodeling in the formation of ventricular trabeculae during early cardiac development.

The functional enrichment of DEGs of ECs/FBs along with the trajectory also revealed that (Figure 5E) HEETG were enriched in the extracellular matrix organization, regulation of endothelial cell proliferation, and endothelial cell proliferation (Figure 5F, Supplementary Table S4), and HEBTG was enriched in fibroblast proliferation, positive regulation of ERK1 and ERK2 cascade, negative regulation of Wnt signaling pathway, stem cell differentiation, and other functions. It should be noted that ERK1 and ERK2 are central molecules to regulate cell division, growth, and survival (Sugden et al., 2011). Similar to CMs and FBs, functional enrichment of DEGs along temporal trajectories also closely reflects the transition of EC/FB populations from immature to mature states. The expression data from e9.5 mECs/FBs corresponded to 10 W hECs/FBs is shown in Figure 5G.

## 4 Discussion

The mice have long been the most widely-used animal model to explore human development and disease. An in-depth comparison between mouse and human heart development at the molecular level is essential for selecting a more appropriate mouse model to focus on human heart development. Currently, cross-species comparisons based on single-cell transcriptomic data have been applied in many organs, such as the liver and bladder (Yu et al., 2019; Wang et al., 2020), whereas the analysis related to cross-species conservation in heart development between human and mouse is not well-established yet. Although a previous work has identified a series of conserved and specific cell type marker genes through compared gene expression profiles between human and mouse, significant limitations are obvious due to the discrete developmental time points of current publicly available mouse datasets in cardiac development and the scarcity of human heart samples (Cui et al., 2019). Integrated analysis of datasets has been widely recognized as one of the effective methods to provide a more comprehensive cross-species comparative analysis. In the present work, we obtained a more comprehensive transcriptomic landscape of mouse heart development by sampling to eliminate biological significance that is difficult to explain due to differences in the number of cells detected and by integrating single-cell transcriptomic datasets from different stages. Then a comparative analysis was performed with the single-cell transcriptome of the human embryonic heart. Our results showed significant lineage-specific changes in the cellular composition and developmental trajectory in both human and mouse fetal hearts. We found that the major cell types and developmental trajectories were broadly similar in the two species. The major cell types, including cardiomyocytes, fibroblasts, endothelial cells/fibroblasts, and immune cells, were successfully identified, and each cell type was shown to be relatively conserved between human and mouse. Through analysis of homologous genes between the two species, we also identified a series of unreported species-shared or species-specific marker genes between major cell types. By constructing developmental trajectories for each major cell type, we obtained a series of genes with similar expression patterns across cell types in human and mouse. Meanwhile, we further detected the most similar time points between human and mouse cell types. These results provide a basis for understudying heart development *via* cross-species comparative analysis.

Cardiac fibroblasts have been confirmed to be formed *via* endothelial mesenchymal transition (EMT), so the signals regulating EMT affect the fate specification of epicardial cells to form fibroblasts (Deb and Ubil, 2014). BMP signaling receptors are highly expressed in fibroblasts during human heart development (Cui et al., 2019), and the pathway is really required for early specification and initiation of EMT (Garside et al., 2013). Here, we divided the genes that affect the developmental trajectory of major cell types into three clusters, which are detected with high expression at the initial, transitional, and terminal stages in the developmental trajectory. We found that the genes expressed in fibroblasts along the end of the proposed trajectory were enriched in the BMP pathway in both human and mouse (Figure 4E), indicating that an active pathway of BMP in fibroblasts is conserved among the species during cardiac development. Moreover, we also observed that the Notch signaling pathway and p53-mediated signaling are mainly involved in the fibroblast-expressed genes at the beginning of the trajectory. Given that Notch signaling can promote EMT and valvular fibroblasts, while P53, as one of the senescence markers, exerts a role in anti-fibrosis by inhibiting cyclin protein, thus blocking the cell cycle for suppressing the proliferation of myofibroblasts (Gao et al., 2020), our results further demonstrated that cardiac development was strictly regulated by the synergistic interactions of different signaling pathways, including BMP, Notch, and p53 signaling pathways, which regulated cardiac development with relatively conserved between human and mouse. In addition, we also identified a series of genes with conserved expression patterns in major cell types of human and mouse hearts, suggesting the most basic cellular makeup in the development.

It has been noted that the temporal evolution of CM gene expression exists in both ventricular and atrial cardiomyocytes in mouse from e9.5 to P21 (DeLaughter et al., 2016). A similar overlapping gradual developmental pattern was also observed in human cardiomyocytes in our work. Using this maturation pattern, we have mapped the temporal expression profile of mouse heart development from e8.5 to e14.5 to human developmental time points, which enabled us to obtain precisely corresponding developmental stage of both mouse and human CMs. We found that the CMs of mouse e9.5 and e10.5 corresponded to that of human 7 W and 9 W, respectively (Figure 3H). In our study with other non-cardiomyocyte subpopulations, such as fibroblasts and endothelial cells, the similar correspondence between mouse and human was also confirmed, respectively. (Figure 4G, Figure 5G). Although we demonstrated the conservation of embryonic heart development between human and mouse by integrating single-cell transcriptome data, more deeper understanding of mammalian heart development is yet to be achieved by integrating multi-omics data or incorporating phylogenetic comparative methods.

In previous works, the analyzed datasets only focused on the molecular mechanisms of single-species heart development. Here, we performed an integrative comparative analysis across species, which revealed unexplored features of lineage-specific changes in cellular composition and developmental trajectories in human and mouse fetal hearts, demonstrating that the gene expression is conserved between lineages of human and mouse hearts. This work is based on bioinformatic analysis and still requires basic experiments to confirm the underlying mechanisms. It is necessary to collect the related multi-omics data with more evidence to clear the conserved and differential heart development mechanisms among different species.

In conclusion, we revealed the evolutionary conservation of key cell populations and molecular features during heart development in human and mouse and also validated related signaling pathways for regulating the development. These results provided a clear frame of the differentiation process of the major cell types in the human and mouse fetal hearts and a theoretical basis for exploring heart development and regeneration using mouse models. Our work also improved the understanding of early cardiac development, suggesting an essential to optimize animal models and *in vitro* cell lineage differentiation when focusing on the human physiological process.

## Data Availability

The original contributions presented in the study are included in the article/Supplementary Material; further inquiries can be directed to the corresponding authors.

## Supplementary Material

Supplementary Figure S1 Quality control, sampling and integration of human and mouse datasets. **(A)** Number of transcripts detected in each cell in the human dataset (GSE106118), red line indicates quality control conditions. **(B)** Number of genes detected in each cell in the human dataset, red line indicates quality control conditions. **(C)** Left: human dataset (GSE106118) doubts filtered; right: the top 2,000 selected highly variable genes. **(D)** Number of transcripts detected in each cell in mouse dataset 1 (GSE76118), red line indicates quality control condition. **(E)** Number of genes detected in each cell in mouse dataset 1, red line indicates quality control condition; **(F)** Left: doublets in mouse dataset 1 (GSE76118); right: doublets in mouse dataset 2 (GSE100861). **(G)** UMAP plots showing sampled mouse dataset 2 (GSE100861) using geosketch after quality control. Left: the UMAP plot before sampling; right: the UMAP plot after sampling, both plots are colored by developmental stage. **(H)** UMAP plots showing the integrated mouse datasets using Harmony. Left: the cell distribution of the mouse datasets before integration; right: the cell distribution of both datasets after integration.

Supplementary Figure S2 Construction of pseudotemporal trajectory of human and mouse cardiomyocytes. **(A)** Heatmap showing the molecular characteristics of each cardiomyocyte subpopulation (human: H, left; mouse: M, right). **(B)** Pseudotemporal trajectory of CM subpopulations constructed by Monocle2 colored by developmental stage (human: H, top; mouse: M, bottom). **(C)** Violin plots showing the expression of LGALS1, ANXA3, SCD in CM subclusters (human: left; mouse: right).

Supplementary Figure S3 Construction of pseudotemporal trajectory of human and mouse fibroblasts. **(A)** Heatmap showing the molecular characteristics of each fibroblast subpopulation (human: H, left; mouse: M, right). **(B)** Pseudotemporal trajectory of FB subpopulations constructed by Monocle2 colored by developmental stage (human: H, top; mouse: M, bottom). **(C)** Violin plots showing the expression of RGS5, BTG2 in FB subclusters (human: left; mouse: right).

Supplementary Figure S4 Construction of pseudotemporal trajectory of human and mouse endothelial cells/fibroblasts. **(A)** Heatmap showing the molecular characteristics of each endothelial cell/fibroblast subpopulation (human: H, left; mouse: M, right). **(B)** Pseudotemporal trajectory of EC/FB subpopulations constructed by Monocle2 colored by developmental stage (human: H, top; mouse: M, bottom). **(C)** Violin plots showing the expression of CD36, FABP4 in EC/FB subclusters (human: left; mouse: right).

Supplementary Table S1 Differentially expressed genes of different cell types in human and mouse.

Supplementary Table S2 Three clusters of affected-trajectory genes in human and mouse based on the developmental trajectory of CMs.

Supplementary Table S3 Three clusters of affected-trajectory genes in human and mouse based on the developmental trajectory of FBs.

Supplementary Table S4 Three clusters of affected-trajectory genes in human and mouse based on the developmental trajectory of ECs/FBs.
