# Supplementary figures and images for: Concordant and Heterogeneity of Single-Cell Transcriptome in Cardiac Development of Human and Mouse
Source: Front Genet. 2022 Jun 27;13:892766. doi: 10.3389/fgene.2022.892766 (PMC9271823; doi:10.3389/fgene.2022.892766)

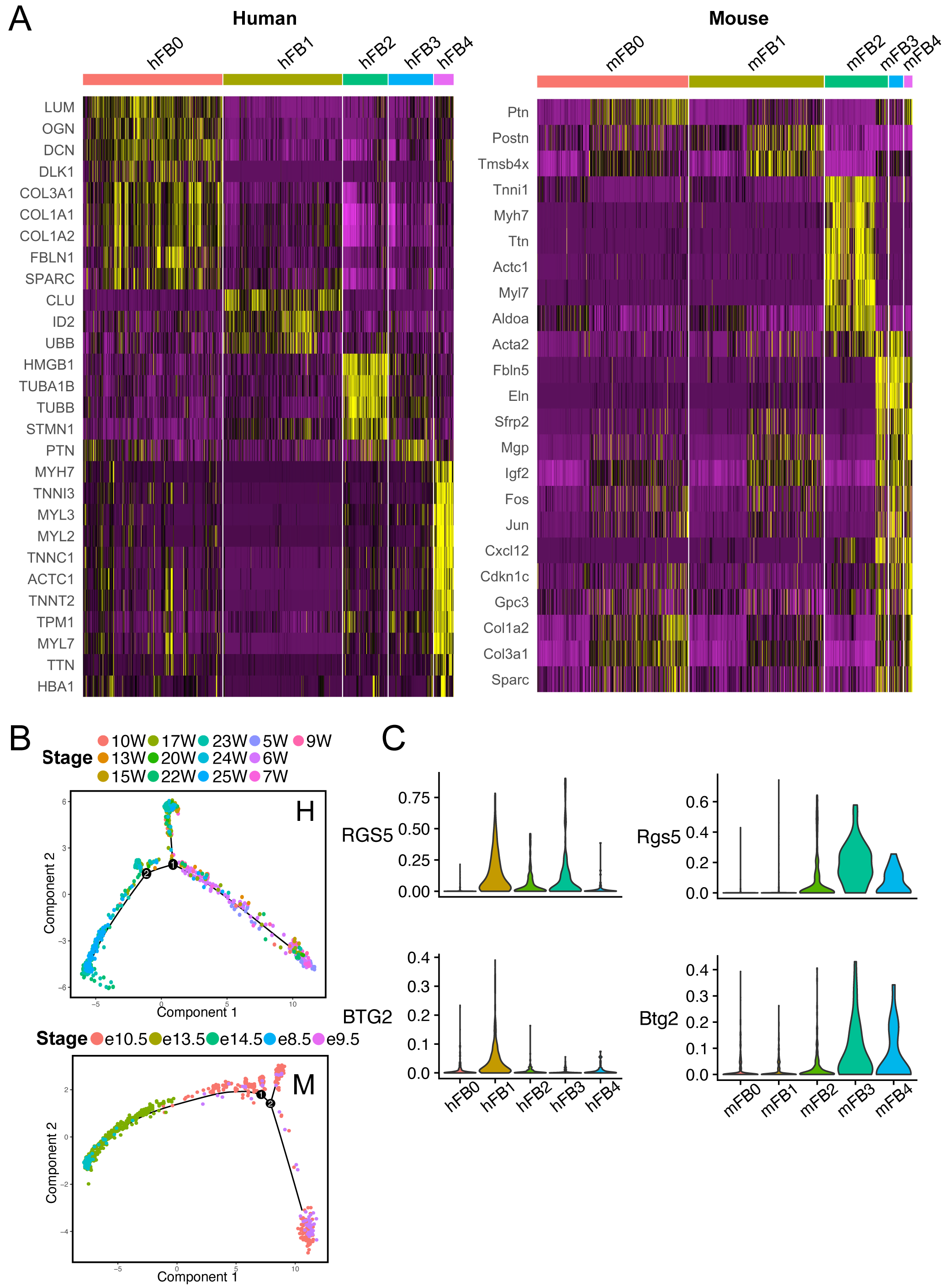

Supplement: Supplementary file 4 [file Image3.TIF]

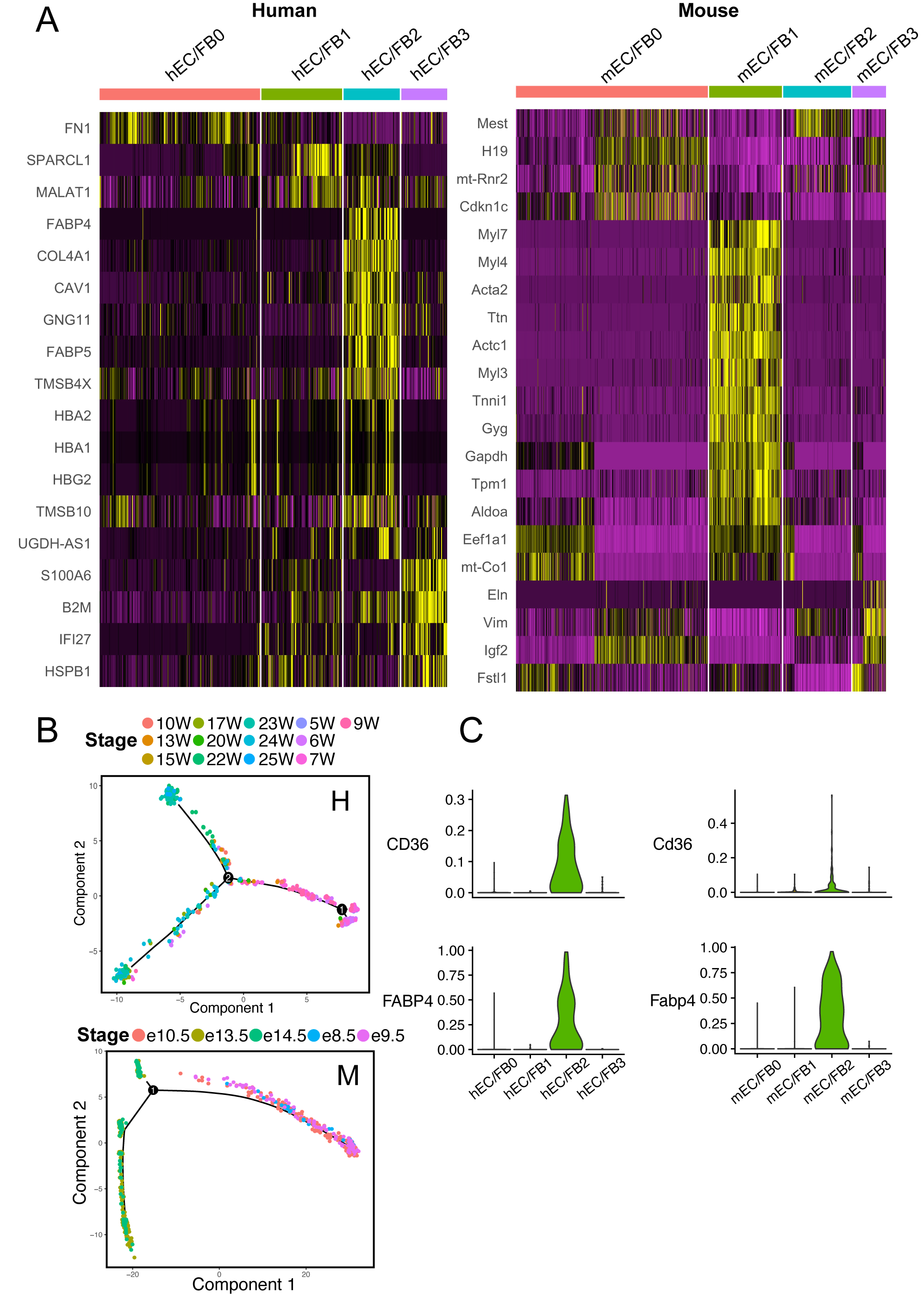

Supplement: Supplementary file 5 [file Image4.TIF]

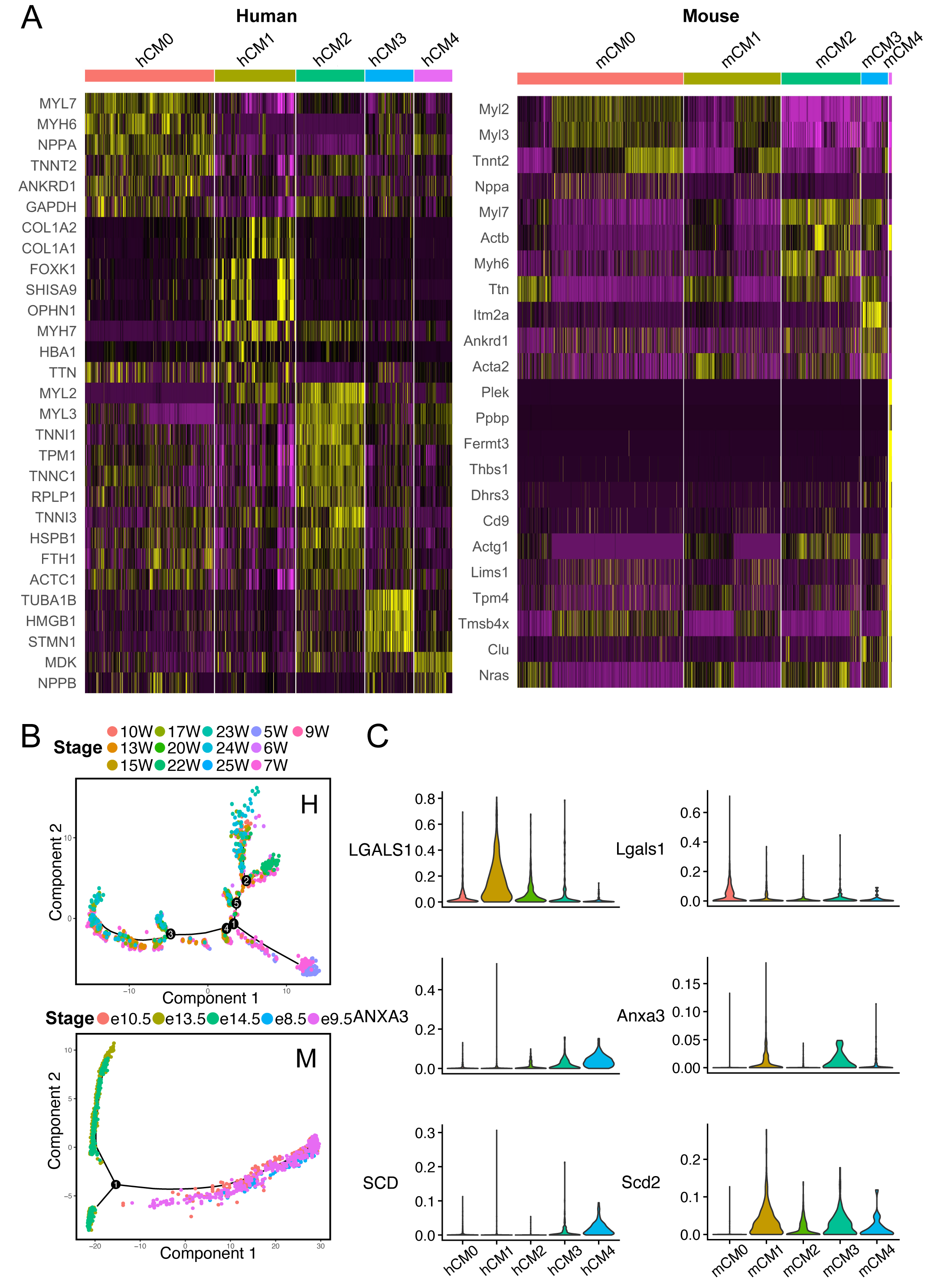

Supplement: Supplementary file 6 [file Image2.TIF]

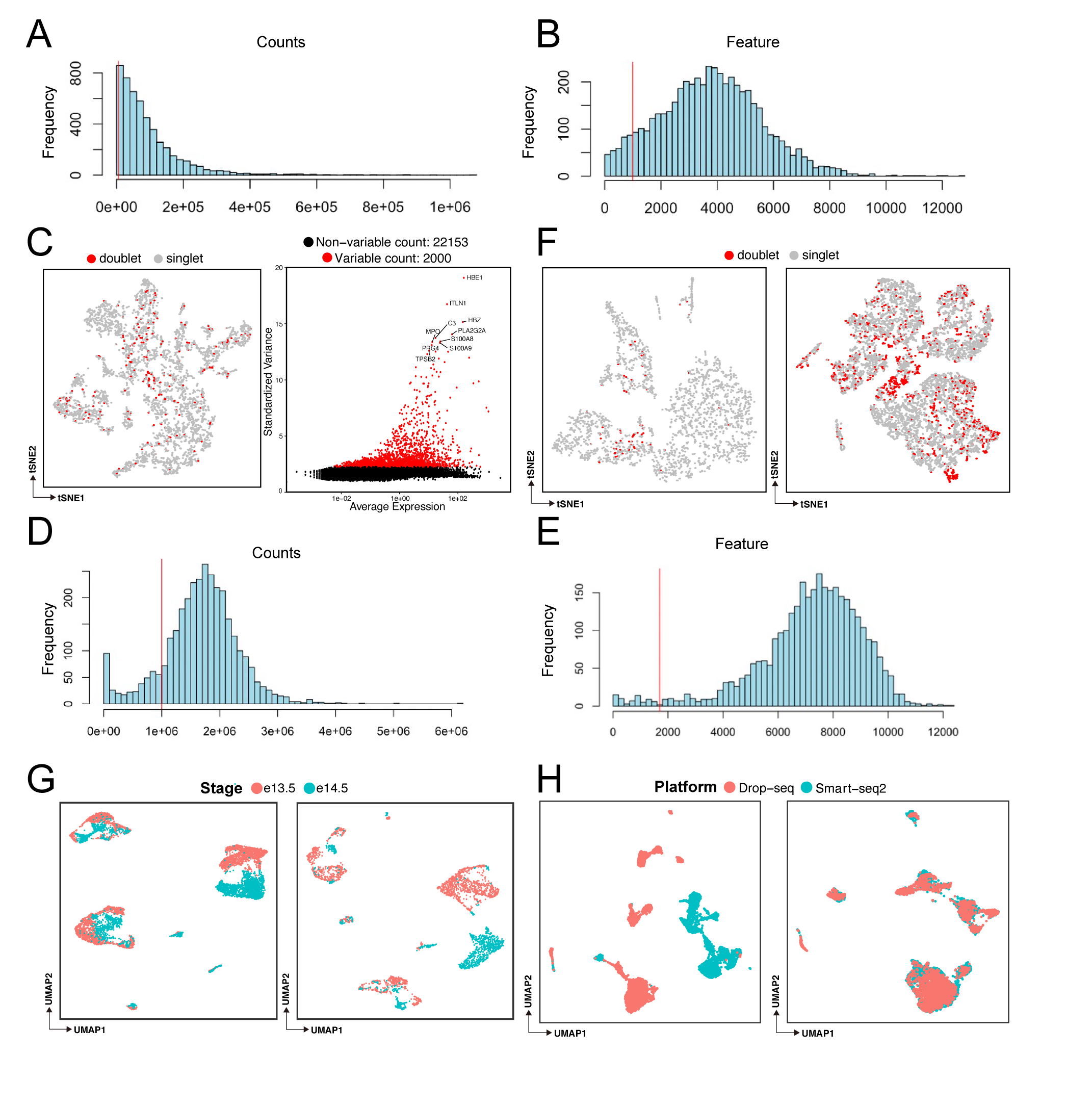

Supplement: Supplementary file 8 [file Image1.TIF]
